# Protein structure and coupled solvent dynamics in α-synuclein fibrils under controlled confinement

**DOI:** 10.1016/j.bpj.2026.01.038

**Published:** 2026-01-29

**Authors:** Katie Lynn Whitcomb, Kurt Warncke

**Affiliations:** 1 Department of Physics, Emory University, Atlanta, Georgia 30322

## Abstract

The formation of amyloid fibrils by the intrinsically disordered protein α-synuclein in human brain neurons and extracellular tissue is a hallmark of Parkinson disease. Although the stable protein β-structure in α-synuclein fibrils has been determined at high resolution, the status of the dynamically disordered domains and their responses to in vivo confinement and small-molecule interactions remain elusive. We use electron paramagnetic resonance spectroscopy of the spin probe, TEMPOL, colocalized with α-synuclein fibrils, and temperature-controlled, ice boundary confinement to characterize the physical and mechanical properties of the dynamically disordered protein and surrounding coupled solvent regions. The spin probe rotational correlation time at all temperature (*T*) values is characterized by two motional components, which are assigned to dynamically disordered protein terminal and hydration phases around the central fibril β-structure. The observed transformation from fast to slow motion components with decreasing *T* and increasing confinement is proposed to proceed through compaction 1 (protein collapse and folding) and compaction 2 (collapse and restriction) processes, intervened by tertiary structure consolidation and stable hydration layer formation. Hysteresis in the return, increasing *T* direction originates from frustration of cooperative dynamics of the spatially overlapping terminal domains and reveals bistability of compact, low-enthalpy and disordered high-entropy structures. Arrhenius and van’t Hoff analyses support the proposed molecular model and indicate that compaction transitions are accessible under physiological conditions. Confinement relief effects of the small molecule dimethylsulfoxide are consistent with the model. The system and results provide a robust platform for assessment of α-synuclein fibril-targeted drugs.

## Significance

The dynamic disorder in α-synuclein and other intrinsically disordered proteins defies high-resolution structural characterization, yet unstructured domains govern physical properties critical for undefined native functions and dysfunction in aggregated states. The 75% proportion of α-synuclein in fibrils that flanks the core cross-β structure is disordered in free solution. Our use of a dynamics-sensitive paramagnetic reporter molecule embedded in the disordered protein-coupled solvent phases, and temperature-modulated ice boundary confinement, identifies a compaction/structure consolidation/compaction sequence in fibrils. Arrhenius and van’t Hoff analyses indicate latent structure formation propensity of disordered α-synuclein domains in fibrils and suggests ordering under in vivo confinement conditions. The system provides a platform for quantitative assessment of single-site and proposed disorder-based ensemble approaches to drug targeting.

## Introduction

α-Synuclein in its monomeric, free-solution form is an intrinsically disordered protein (IDP; 140 amino acids, 14.5 kDa) (1,2), which is associated with the processes of intracellular neurotransmitter trafficking, release, and retrieval from the synaptic cleft in brain neurons (3,4,5). Aggregate oligomer and fibrillar forms of α-synuclein are a hallmark of Parkinson disease and other amyloid pathologies in humans and animal models (6,7). Single-filament protofibrils (∼5 Å diameter) of α-synuclein have a β-sheet core formed from the central nonamyloid-β component (NAC, resides 61–95) and the distal residues (∼30–60) of the N-terminal domain (NTD, residues 1–60; net positive charge) (8,9). Protofibrils combine through cross-β interaction to form mature fibrils (∼10 Å diameter) (10). The dynamically disordered C-terminal domain (CTD, residues 96–140; net negative charge and proline kinks in pseudo-repeat sequence), as well as the disordered proximal NTD, both project from the β-sheet core of the stacked, α-synuclein monomers along the fibril axis. Using controlled, low-temperature (*T*) confinement, we have found that monomeric (11) and oligomeric and fibrillar (12) α-synuclein display the common, dual property of volume compaction with maintained, robust dynamic disorder in the noncompacted protein and surrounding, coupled solvent (12). Here, we address the ensemble properties and detailed molecular mechanism of protein and coupled solvent structural reorganization in α-synuclein fibrils under controlled confinement.

**Figure 1:**
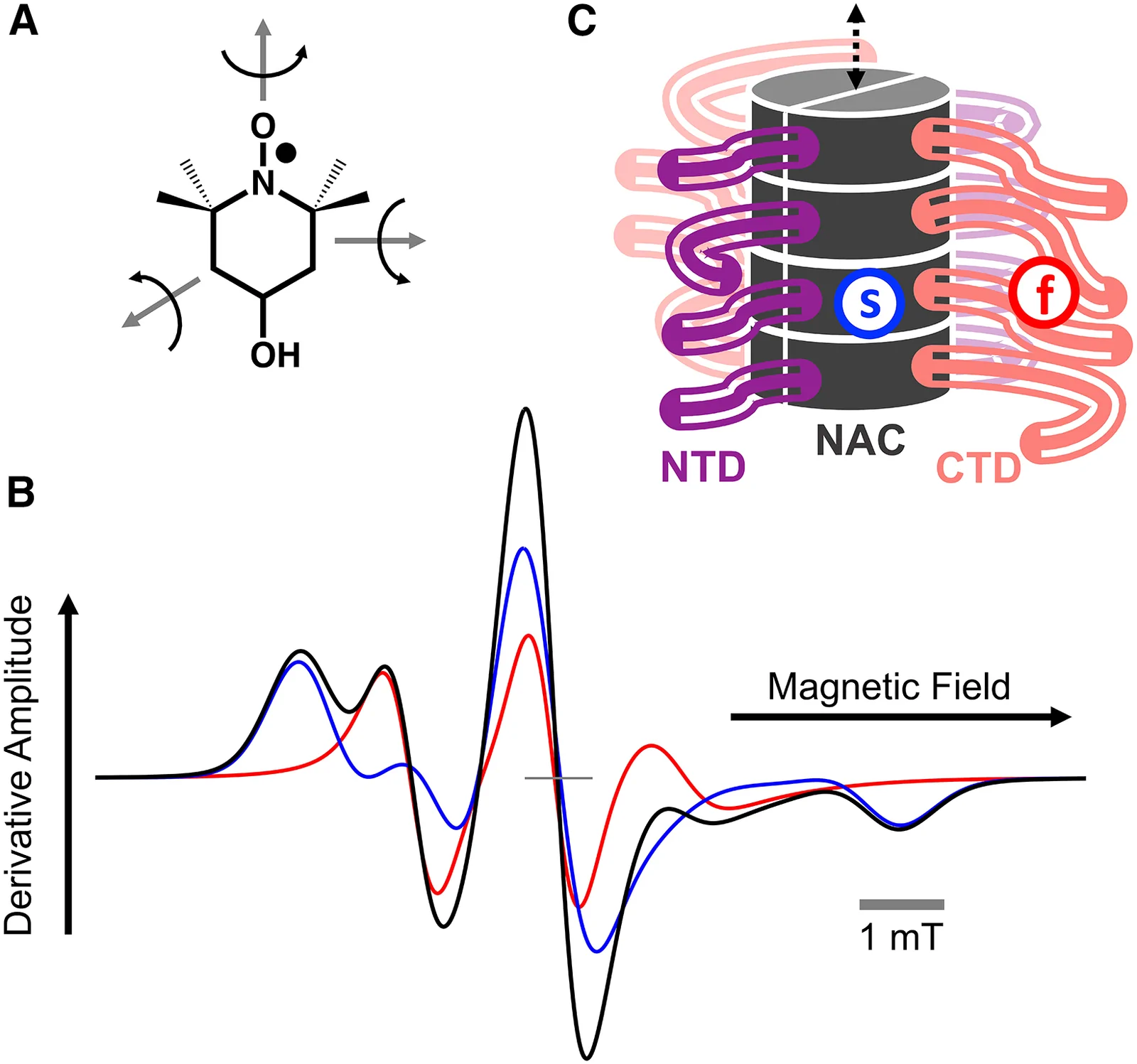
Correspondence of EPR spin probe motional properties and protein structure and dynamics. (*A*) Depiction of TEMPOL spin probe and rotational components about Cartesian axes. (*B*) Two-component TEMPOL EPR spectrum and overlaid simulation, showing slow (*blue*) and fast (*red*) motional components and their sum (*black*; represents the observed spectrum). Parameters represent α-synuclein fibril sample at *T* = 230 K. Zero-crossing of central derivative feature (*horizontal bar*) corresponds to *g* = 2.01. (*C*) Depiction of protein-associated solvent phases and colocalized TEMPOL (represented by circles: slow (*s*, *blue*) and fast (*f*, *red*) components of rotational motion) around the α-synuclein fibril.

Confinement, or the effect of excluded volume owing to an impenetrable boundary (13), offers resolution and quantification of the mechanism and thermodynamics of compaction and inferences about underlying microscopic protein and solvent structure and dynamics. Low to moderate levels of compaction with maintained structural disorder have been demonstrated for α-synuclein under different conditions of the related effect of crowding (effect of excluded volume arising from a high total volume fraction of macromolecules (13)) in vitro (14) and in biological cells (15,16,17), and they are a general property of IDPs (18). We introduce confinement through the ice boundary that forms around the protein in a low-temperature (*T*), frozen solution system and control it by varying *T* over the wide range of 200–265 K (19,20,21,22). During sample freezing, solutes are excluded from the advancing ice fronts, leading to co-residence with protein in interstitial regions within the polycrystalline bulk ice. Inclusion of the electron paramagnetic resonance (EPR) spin probe, TEMPOL (Fig. 1 *A*), leads to its localization in the solvent phases associated with the protein. TEMPOL reports on the protein-coupled solvent dynamics, through its rotational correlation time (*τ*_c_). All α-synuclein forms show two TEMPOL motional spectral components (Fig. 1 *B*) (11,12), designated slow (*τ*_c,s_; location in nominally stable, protein hydration regions) and fast (*τ*_c,f_; location in dynamically disordered regions). The fast, *τ*_c,f_, component is dominant at high *T* values, but its amplitude (or weight, *W*_f_) decreases with decreasing *T*, concomitant with an increase in the slow component (*τ*_c,s_, *W*_s_). This distinctive confinement dependence of α-synuclein arises from compaction of disordered domains (proportion represented by *W*_f_) to form less dynamic protein structure (represented by *W*_s_) (Fig. 1 *C*). In monomeric α-synuclein, we have shown that compaction proceeds in two stages with decreasing *T*, assigned to formation of tertiary structure around the distal-NTD/NAC core (compaction 1 process), followed by collapse of the disordered terminal domains (CTD, NTD) around this framework (compaction 2) (11). The resulting detailed molecular model (11) for the response of monomeric α-synuclein to confinement is informed by and consistent with other experimental (15,23,24,25) and computational (23,24,26,27,28,29) studies.

The molecular structure of α-synuclein in protofibrils and fibrils has been determined by techniques of solid-state nuclear magnetic resonance (NMR) (8,9) and cryo-electron microscopy (cryo-EM) (30,31,32,33). The central domain from residues 37 ± 1 through 97 ± 2 is involved in a back-folded, Greek key, β-strand motif in protofibrils, which stacks to form the β-sheet, and subsets of these residues are involved in the cross-β interaction of two protofibrils that form the fibril. Within this general fibril architectural theme, differences in the Greek key structure and cross-β contacts define several fibril polymorphs (10). Notably, the NTD and CTD regions outside of the core ∼37–97 region are not included in the structures, because of their static (NTD) or dynamic (CTD) disorder (8,9). Our approach of spin probe EPR and controlled confinement is of value because it is unique in assessing the dynamical properties of the terminal domains and their associated solvent and for inferring the ensemble structural features of these flanking regions and their relation to the core protein segments. Here, we advance our previous study (12), by resolving confinement-induced compaction 1, compaction 2, and intervening tertiary/quaternary structure consolidation processes in the fibrillar form of α-synuclein, with quantification by thermodynamic (van’t Hoff) and kinetic (Arrhenius) analysis. The results, including the variation of confinement by using dimethylsulfoxide (DMSO) (20,22) and determination of the origin of the thermal hysteresis in the coupled protein and solvent organization upon compaction and decompaction, lead to a comprehensive model for α-synuclein in fibrils. The system and approach are relevant to identifying and quantifying recently proposed entropically based (34) and ensemble (35) paradigms for drug targeting.

## Materials and Methods

### EPR sample preparation

All chemicals were obtained from commercial sources. Deionized water was used (resistivity, 18.2 MΩ·cm; Nanopure system, Siemens). Preformed fibrils of human α-synuclein (UniProt, P37840; National Center for Biotechnology Information (NCBI), gene SNCA) were obtained from rPeptide (Watkinsville, Georgia, US; P/N S-1001-2) as a suspension (10 mg/mL). Fibrils are 50–200 nm in length and approximately 10 nm in width, as shown by transmission electron microscopy (12), consistent with the cross-β interaction of two β-sheet-structured protofilaments. The standard EPR sample (designated control) contained 18.4 μM α-synuclein in fibrils, 0.02 mM TEMPOL, and 10 mM potassium phosphate buffer (pH 7.4), in a final volume of 0.3 mL. EPR samples for experiments with varied DMSO concentration contained added 0.033 (1×) and 0.099 (3×) % *v*/*v* DMSO. The molar ratio of DMSO to α-synuclein in the 1× sample was 260:1. Samples were transferred to EPR tubes (4 mm outer diameter; Wilmad-LabGlass, Buena, NJ, US; P/*N* 707-SQ-250M), frozen by immersion in isopentane at 140 K, and stored in liquid nitrogen before measurements (22).

### Continuous-wave EPR spectroscopy

X-band CW-EPR spectroscopy was performed by using a Bruker E500 ElexSys EPR spectrometer and ER 4123SHQE X-band cavity resonator with temperature calibration and control, as described (36). The followings acquisition parameters were used: microwave frequency, 9.5 GHz; microwave power, 0.2 mW; magnetic field modulation, 0.2 mT; and modulation frequency, 100 kHz. Four spectra were averaged at each temperature.

### EPR line shape and EPR simulations

The EPR spectrum from the spin probe, TEMPOL, arises from the interactions of the unpaired electron spin (*S* = 1/2) with the external magnetic field (Zeeman interaction; defined by the **g** tensor) and the nitroxide ^14^N nuclear spin (*I* = 1) through the hyperfine interaction, which is defined by the ^14^N hyperfine tensor. The three spectral features correspond to electron spin transitions (Δ*m*_s_ = ±1/2) among the three ^14^N nuclear spin states, *m*_I_ = 0, ±1 (37). At higher temperatures, averaging of the dipolar hyperfine interaction by relatively rapid rotational motion of the spin probe resolves three spectral features, which are separated by the isotropic ^14^N hyperfine coupling constant. Slower rotation at lower temperatures leads to manifestation of the orientation-dependent dipolar hyperfine interaction, which broadens each *m*_I_ feature. The rotational correlation time, *τ*_c_, and component weights, *W*, which characterize the motional effects on the TEMPOL line shape (38,39), are determined by simulation of the EPR spectra. Methods for the simulation of nitroxide EPR spectra in the low-*T* frozen solution system, by using the EasySpin (40) toolbox in the MATLAB (The MathWorks, Natick, MA) software environment, and a common set of **g** and ^14^N hyperfine tensor principle values, have been described in detail (22). The correlation times obtained from the simulations are in excellent agreement with those calculated for known solvent viscosity by using the Debye-Stokes-Einstein expression (41) and correspond to slow (*τ*_c_ →10^−7^ s), intermediate, and rapid (*τ*_c_ →10^−11^ s) TEMPOL tumbling regimes that define the X-band motion-detection bandwidth (38).

### Temperature dependence of spin probe rotational correlation time

The *T* dependence of the rotational correlation time was addressed by using the molar form of the expression from Arrhenius rate theory (42):

(Equation 1) $$\tau_{c}=\tau_{c,0}\;\exp\left(\frac{E_{a}}{RT}\right)\text{,}$$

where *τ*_*c*,0_ (units, seconds), *E*_*a*_ (kcal/mol), and *R* (1.987 cal/mol/K) are the Arrhenius rotational correlation time prefactor, activation energy, and gas constant, respectively. The expression implies that *E*_a_ and *τ*_c,0_ are *T* independent over the measurement range. In this case, plots of log*τ*_c_ versus *T* ^−1^ yield a linear relation with slope $\frac{E_{a}}{R}$ and vertical axis intercept of ln(*τ*_c,0_).

### Temperature dependence of spin probe mobility portioning between phases

Two mobility components are labeled as slow (s; relatively slow motion, larger *τ*_c_ value) and fast (f; relatively fast motion, smaller *τ*_c_ value), distinguished by different spin probe *τ*_c_ values. The relative amplitudes of the spin probe rotational mobility components represent the relative volumes of the distinct solvent phases, in which the spin probe resides (20,22). The amplitudes, or weights, *W*, are normalized, so that the sum of the slow and fast weight components, *W*_s_ and *W*_f_, at each *T* value is unity. We use van’t Hoff analysis, as described previously (11), to determine the Gibbs free energy difference for the direction of fast to slow solvent component conversion:

(Equation 2) $$\Delta G=-2.3RT\;\log\left(\frac{W_{s}}{W_{f}}\right)$$

When enthalpy (Δ*H*; kcal/mol) and entropy (Δ*S*; cal/mol/K) are *T* independent, plots of $\log\left(\frac{W_{s}}{W_{f}}\right)$ versus *T* ^−1^ yield a linear relation, with slope of $-\frac{\Delta H}{2.3R}$ and vertical axis intercept of $\frac{\Delta S}{2.3R}$ (42).

## Results

### Temperature dependence of the TEMPOL EPR spectrum under varied dimethylsulfoxide concentration

**Figure 2:**
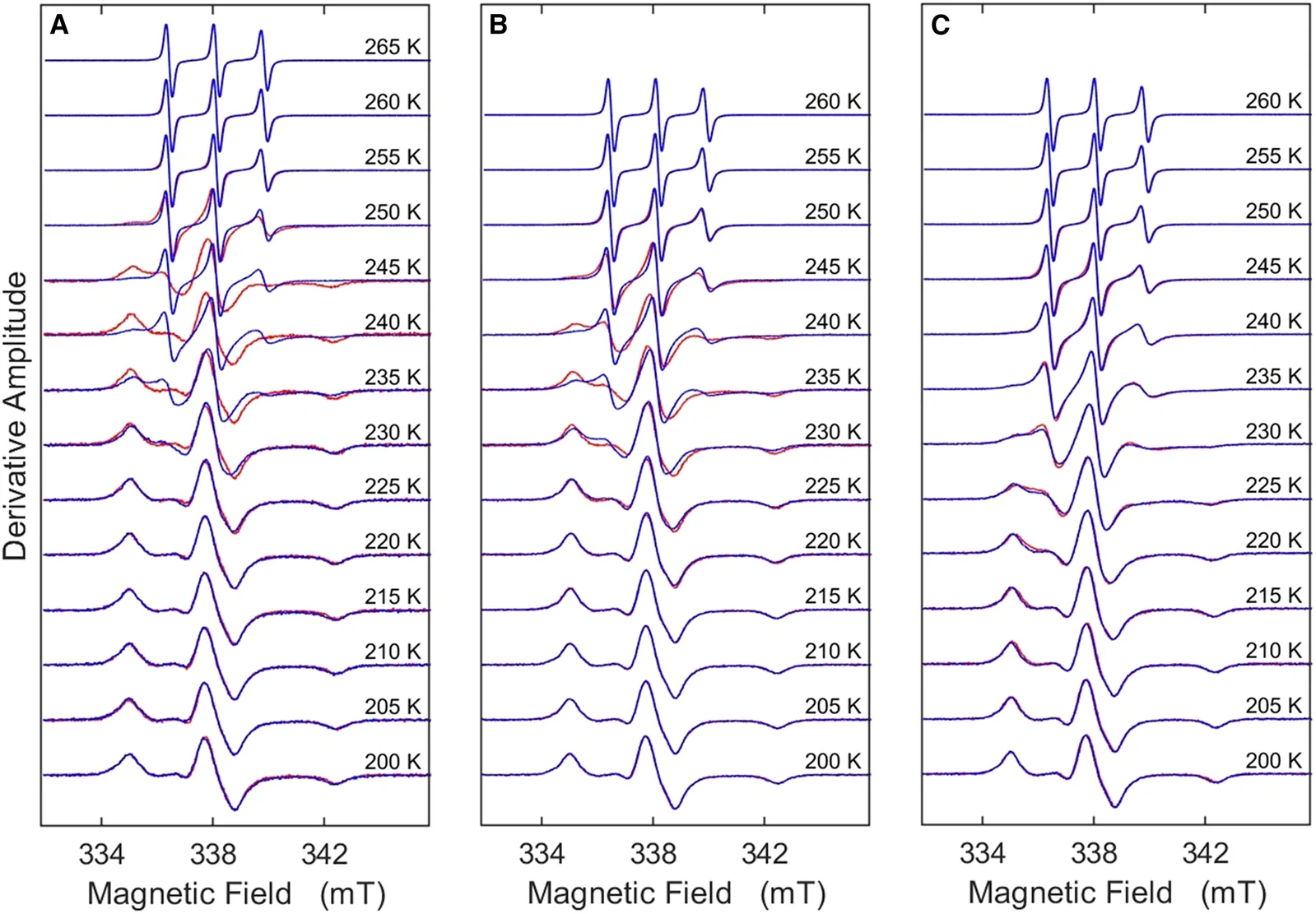
Temperature dependence of the TEMPOL EPR spectrum for fibrillar α-synuclein samples. (*A*) no DMSO (control), (*B*) 1× added DMSO, (*C*) 3× added DMSO. Spectra in increasing *T* direction (*red*) and decreasing *T* direction (*overlaid, blue*) are normalized to the central peak-to-trough amplitude.

In the low-*T* system with folded, globular proteins, DMSO is excluded (43,44) from the protein hydration layer, forming a surrounding aqueous-DMSO eutectic phase that maintains *T*-independent phase proportion (*W*_f_) and log*τ*_c,f_ values over a wide range (19,20). We hypothesized, based on our previous model (12), that DMSO added to the α-synuclein system would infiltrate the disordered and unstable hydration solvent phases, a dramatically different effect relative to globular proteins. DMSO addition might also provide insight into the degree of protein control of the dynamically disordered phase. Thus, we added DMSO at two concentrations, 1× (260:1 molar ratio of DMSO to α-synuclein) and 3× (780:1), to the standard (control) fibrillar α-synuclein sample. TEMPOL spectra were measured sequentially, in 5-K increments, in increasing and then decreasing *T* directions between 200 and 265 K (Fig. 2). In the decreasing *T* direction of measurement, spectra evolve characteristically from a motionally narrowed line shape at elevated *T* to a rigid-limit, broad powder pattern at low *T*. The qualitatively similar trends in evolution of the line shapes for the samples (Fig. 2) with decreasing *T* value indicate decreasing motion in the protein-coupled solvent in which TEMPOL resides. Measurements in the direction of increasing *T* indicate a return of protein-coupled solvent to the more mobile state.

### Temperature dependence of TEMPOL rotational correlation times and component weights under different dimethylsulfoxide concentrations

**Figure 3:**
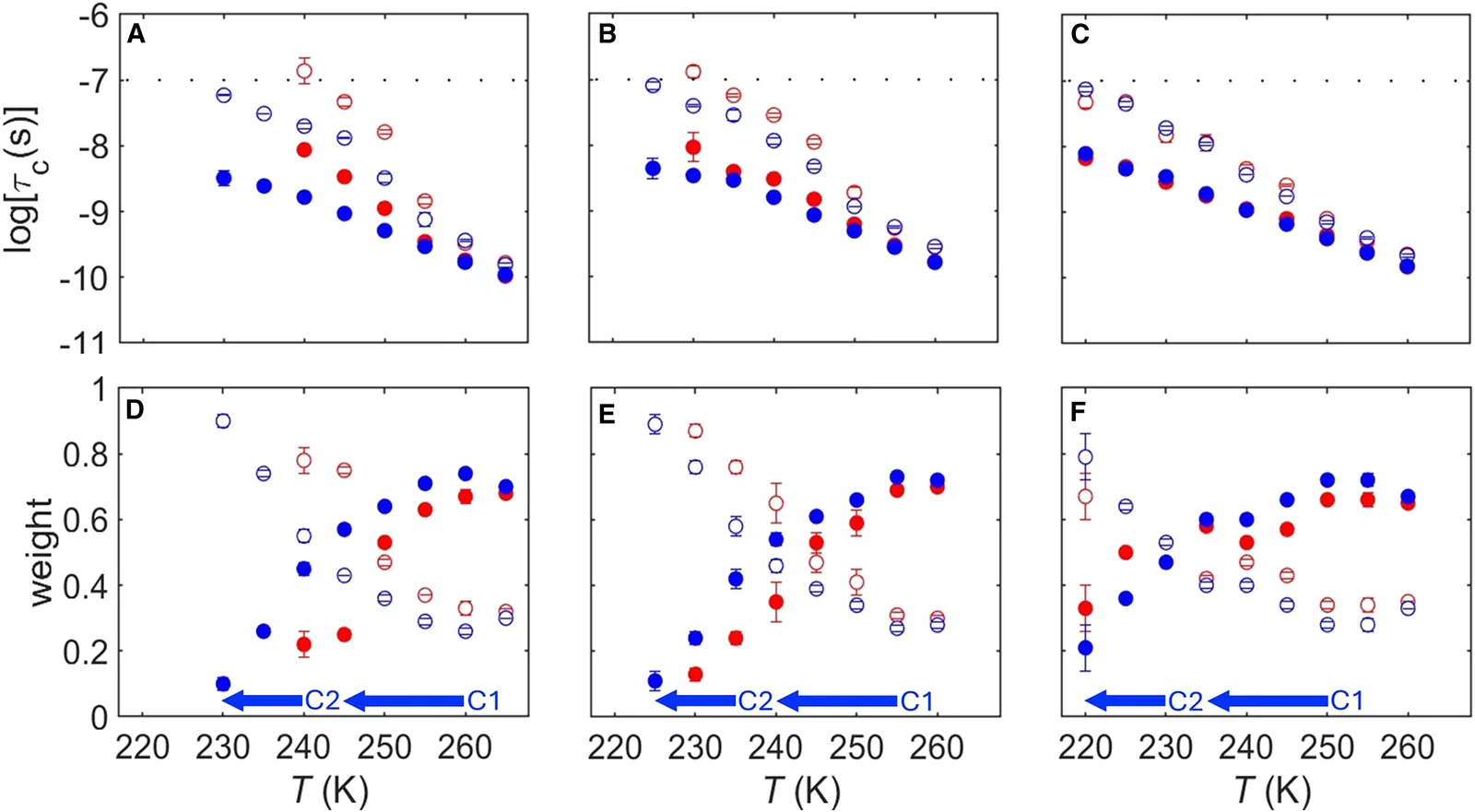
Temperature dependence of the simulated rotational correlation time and normalized mobility component weight of the standard fibrillar α-synuclein sample. (*A*, *D*) No added DMSO, 0×; (*B*, *E*) 1× added DMSO; (*C*, *F*) 3× added DMSO. Open circles represent the slow component (log*τ*_c,s_, *W*_s_) and solid circles represent the fast component (log*τ*_c,f_, *W*_f_) for the increasing (*red*) and decreasing (*overlaid, blue*) *T* directions of spectrum acquisition. Regions of compaction 1 and 2 for decreasing *T* are identified by blue arrows, labeled as C1, C2. Error bars indicate the standard deviations from two independent data sets. Reference value for *τ*_c_ is 1 s. The dotted line at log*τ*_c_ = −7 displays the mobility detection upper limit.

The EPR line shapes of TEMPOL at increasing DMSO concentration are reproduced by spectral simulations at each *T* value by using two rotational mobility components, relatively slow (log*τ*_c,s_, *W*_s_) and fast (log*τ*_c,f_, *W*_f_) (Fig. S1, overlaid experimental and simulated spectra; Tables S1 and S2, simulation parameters). When mobility is expressed as log*τ*_c_, the argument of the logarithm is referenced to the value, *τ* = 1 s. Focusing on the data for the direction of decreasing *T* (blue data, Fig. 3), the control and added DMSO conditions all display a two-stage decrease in *W*_f_, indicating that, as for monomeric α-synuclein (11), fibrillar α-synuclein displays the compaction 1 and compaction 2 processes with decreasing *T* and increasing ice boundary confinement. In comparison with control, DMSO at 1× primarily influences the *W* values (increase in *W*_f_ at each *T* value) in the compaction 2 process. Interestingly, the log*τ*_c,f_ values of 1× DMSO and control are comparable over the full *T* range. Addition of DMSO at 3× leads to significant changes in log*τ*_c,s_ and *W*_f_ in the compaction 2 region (equivalent to approximately −5 K shifts in the *T*-dependence), while preserving log*τ*_c,f_ across the *T* range, and with a smaller effect on the compaction 1 parameters. Fig. 3 also shows that the thermal hysteresis, or separation of log*τ*_c_ and *W* parameters for increasing (red data) and decreasing (blue data) directions of *T*, narrows with 1× DMSO and is further reduced at 3× DMSO. We used circular dichroism (CD) spectroscopy to confirm the stability of the α-synuclein fibrils in the presence of DMSO, by showing the maintenance of their characteristic secondary structure signature (Fig. S2). Overall, DMSO addition leaves intact the general trend of two compaction processes under confinement (11) and maintains the characteristic, two-component log*τ*_c_ and *W* behavior. This suggests that DMSO infiltrates the protein-coupled solvent regions, rather than forming a separate, dynamically distinct, aqueous-DMSO eutectic phase, as for globular proteins (20,41).

### Influence of temperature cycling range on thermal hysteresis

**Figure 4:**
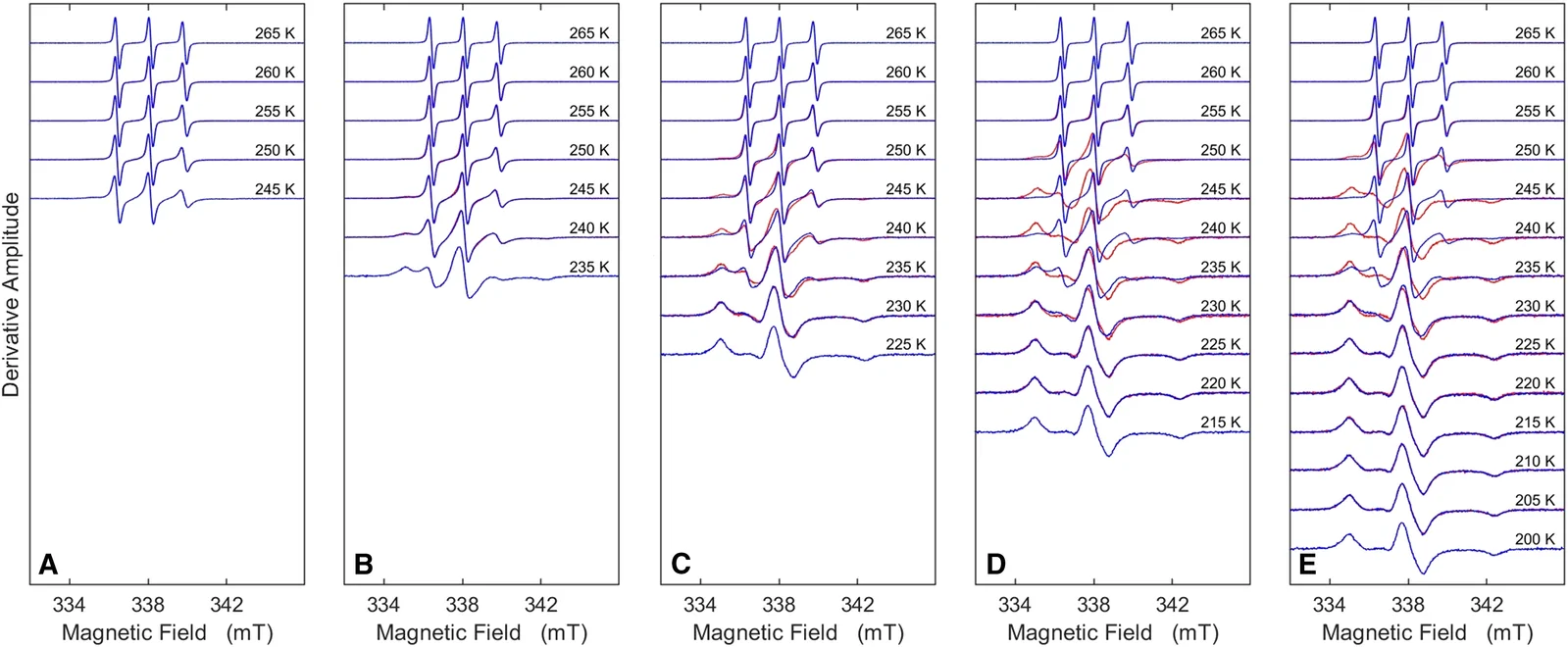
Temperature cycling dependence for the TEMPOL EPR spectra of fibrillar α-synuclein. Spectrum cycles start from 265 K with spectrum collection at 5-K increments in the direction of decreasing *T* (*blue*) and decrease to a minimum *T* stop point at (*A*) 245 K, (*B*) 235 K, (*C*) 225 K, (*D*) 215 K, and (*E*) (200 K). Spectra are then collected at the same *T* values in the direction of increasing *T* (*red*), returning to 265 K. Spectra for *T* decrease are overlaid on spectra for *T* increase. Spectra are normalized to the central peak-to-trough amplitude.

**Figure 5:**
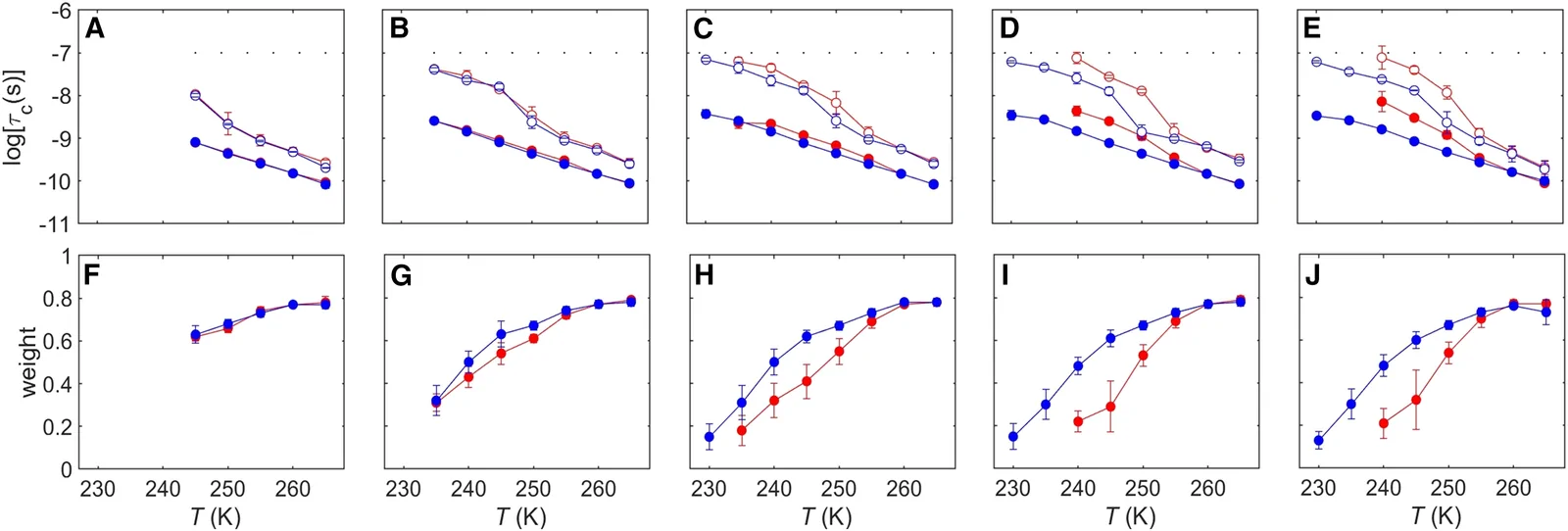
Temperature cycling dependence of the simulated rotational correlation time and normalized fast mobility component weight of control fibrillar α-synuclein. Parameters in plots of log*τ*_c_ (log*τ*_c,s_, *open circles*; log*τ*_c,f_*solid circles*) and *W*_f_ correspond to the spectra in Fig. 4. Cycles start from 265 K with spectrum collection in the direction of decreasing *T* (*blue*) and decrease to a minimum *T* stop point before return to 265 K with increasing *T* (*red*). (*A*, *F*) 245 K, (*B*, *G*) 235 K, (*C*, *H*) 225 K, (*D*, *I*) 215 K, and (*E*, *J*) 200 K. Lower limit *T* of 230 K in the plots corresponds to bandwidth limitation (rigid limit, log*τ*_c_ < 7.0; *dashed line in A–E*) on the simulation of the TEMPOL spectra. Error bars indicate the standard deviations from two independent data sets. Reference value for *τ*_c_ is 1 s.

To resolve the *T* range of hysteresis inception and development, the directional *T* protocol was applied to α-synuclein (absence of DMSO), but the *T* range was cycled sequentially from a high *T* of 265 K to lower limit values of 245, 235, 225, 215, and 200 K, followed by return from each lower limit *T* value to 265 K (depiction, Fig. S3). The decreasing *T* spectra are identical for each cycle, as expected, but the return, increasing *T* direction spectra show different degrees of hysteresis (Fig. 4). The overlaid simulation mobility parameters for data collected in the direction of decreasing and increasing *T*, for each lower limit *T* value, are shown in Fig. 5 (spectrum-simulation overlays, Fig. S4; Table S4, simulation parameters). Fig. 5 shows that hysteresis in both log*τ*_c_ and *W* parameters is absent in the 245 K lower limit cycle, grows in extent for 235 and 225 K, and displays a comparable degree for 215 and 200 K. These results indicate that the process that causes hysteresis in TEMPOL rotational motion and component weights develops incrementally and cumulatively over only the lower-*T* range, *T* < 245 K.

## Discussion

### Two distinct compaction transformations characterize the response of fibrillar α-synuclein to increasing confinement

**Figure 6:**
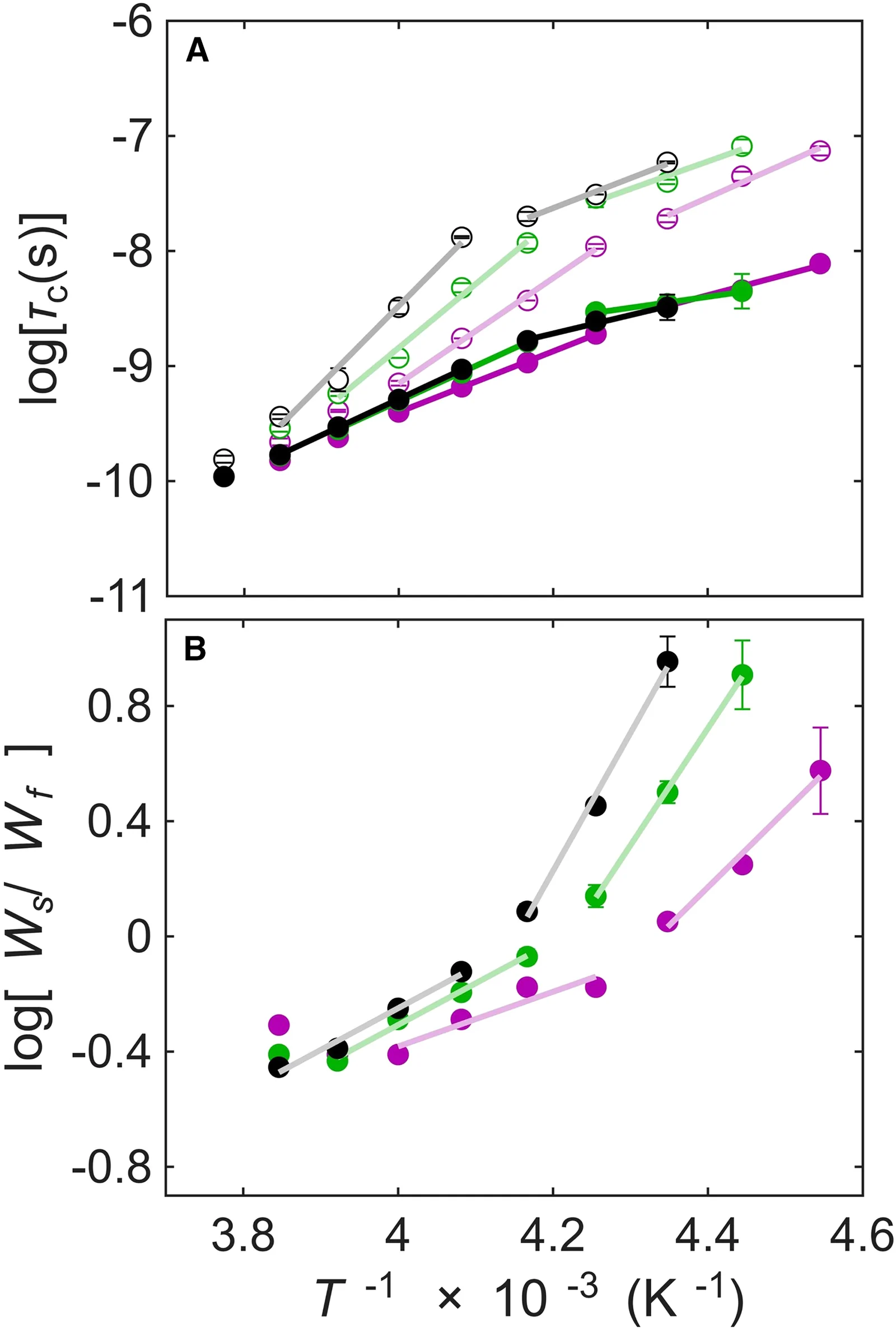
Dependence of the rotational correlation time and ratio of normalized slow and fast mobility component weights as log_10_ value of TEMPOL on *T*^−1^ for control and added DMSO. (*A*) Arrhenius plot of rotational correlation time, as log*τ*_c_ (log*τ*_c,s_, *open symbol*; log*τ*_c,f_, *solid*). (*B*) van’t Hoff plot of normalized weight ratio, as log(*W*_s_/*W*_f_). Results for control (no DMSO, *black*) are overlaid with 1× DMSO (*green*) and 3× DMSO (*violet*). Results for the decreasing direction of sequential temperature change are shown. Solid lines correspond to linear fits to the indicated regions (fitting parameters, Table S5). Error bars represent standard deviations from two independent data sets. Reference value for *τ*_c_ is 1 s.

To quantify the effects of ice boundary confinement on α-synuclein fibril structure and dynamics, and its modulation by DMSO and protein concentration, the *T* dependences of the spin probe mobility parameters were addressed by using the Arrhenius and van’t Hoff formalisms (Eqs. 1 and 2). Fig. 6, *A* and *C* shows the *T* ^−1^ dependence of log*τ*_c_ and log(*W*_s_/*W*_f_) values for α-synuclein (absence of DMSO), for spectrum acquisition in the direction of decreasing *T* (fitting parameters, Table S5). The plots resolve distinct linear high *T* and low *T* regions in the log(*W*_s_/*W*_f_), log*τ*_c,s_, and log*τ*_c,__f_ dependences, corresponding to the compaction 1 and 2 processes in Fig. 3, *A* and *D*, which are delineated by a bend, or kink. This general trend is preserved under all of the conditions, suggesting that the same basic molecular mechanism prevails. In general, linear *T* ^−1^ plots indicate constant *E*_a_ and log*τ*_c,s,0_ (Eq. 1) and Δ*H* and Δ*S* (Eq. 2) parameters. Thus, the parameters obtained from the linear fits in Fig. 6 are constant over the ranges of the relations to within the limits specified by their standard deviations (Table S5). Therefore, over the range of these linear regions, the protein hydration and dynamically disordered solvent phases, represented by the slow and fast spin probe mobilities, maintain their physical properties. The *T*-independent log(*W*_s_/*W*_f_) at the highest *T* values (Fig. 3; no DMSO, *T* ≥ 260 K; 1× DMSO, *T* ≥ 255 K; 3× DMSO, *T* ≥ 250 K) suggests a low-confinement condition that precedes compaction initiation. Surprisingly, the fibril log*τ*_c,s_ and log*τ*_c,f_ relations have a lower *E*_a_ (shallower slope) in the low-*T*, compaction 2 regime. A comprehensive model for the confinement response of α-synuclein fibrils must incorporate all of the above features and, in addition, account for the hysteresis and the effects of DMSO.

### Model for fibrillar α-synuclein under temperature-controlled confinement

**Figure 7:**
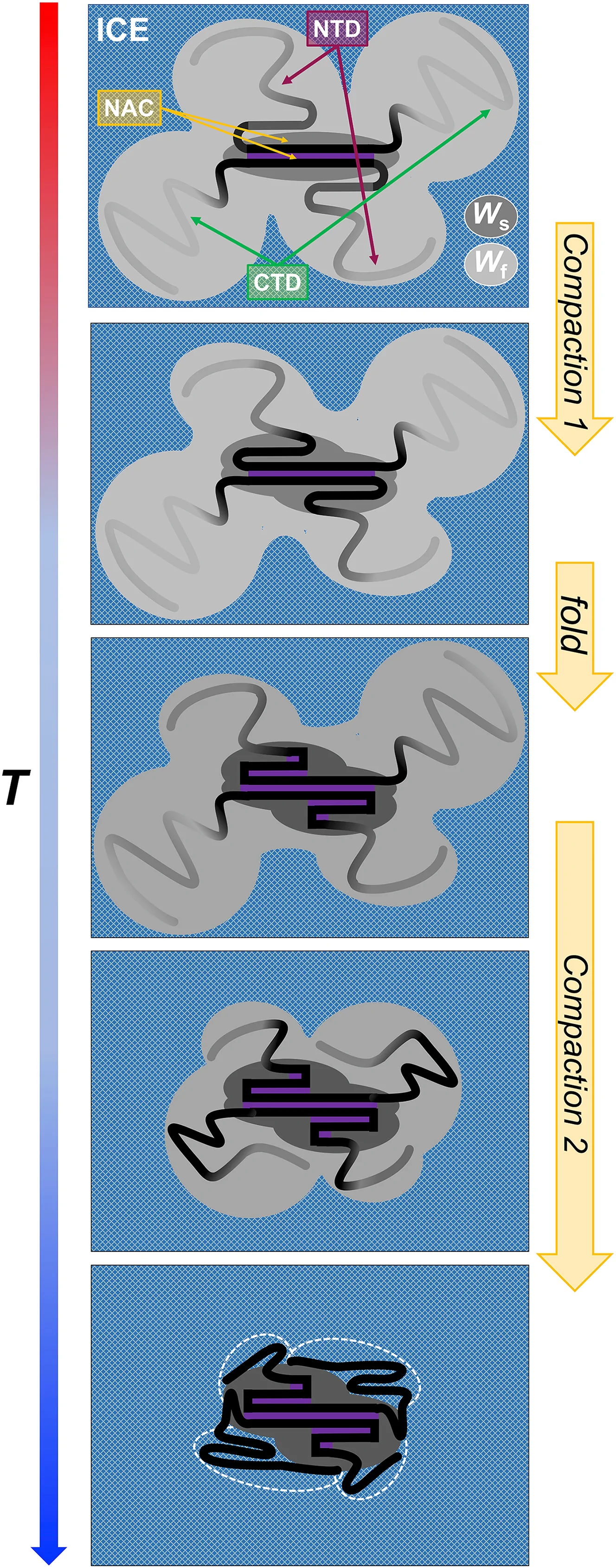
Model for α-synuclein fibrils under *T*-controlled ice boundary confinement. The view is a transverse section through the fibril, showing one layer of two α-synuclein monomers, linked by the cross-β interaction. The fibril axis runs into and out of the plane of the page. The depiction of the structure, dynamics, and events corresponds to decreasing *T*, starting from high *T* (*red shading in temperature indicator arrow*) to low *T* (*blue shading*). α-Synuclein protein domains, ice, and hydration (corresponding to spin probe-reported *W*_s_; *darker gray at each T value*) and dynamically disordered (*W*_f_; *lighter gray*) solvent are defined in the top panel. Protein is depicted as thick lines. Stable protein domains are black, fading to lighter gray in proportion to increased dynamic disorder. Black, rounded loop regions show relatively compacted, developing protein structure, and rectilinear regions represent stable tertiary structure, with violet interaction regions. Bottom panel at lowest *T* value depicts regions occupied by statically disordered protein (*white dashed boundaries*).

Our model for the molecular mechanism of confinement-controlled α-synuclein fibril compaction and expansion is represented by the progression of states depicted in Fig. 7. At high *T* values, the cross-β regions resolved by NMR and cryo-EM studies (8,10,45) anchor the protruding disordered CTD and NTD segments. Interlayer interactions of the terminal domains create an extended dynamically disordered, protein-coupled solvent region along the fibril axis. Decreasing *T* favors progressive net movement of fluid water from within the protein domains to the ice boundary, where the water crystallizes, inwardly growing the ice boundary, which leads to exclusion of the volume accessible to the protein. As *T* is lowered, we propose that compaction of the protein first occurs primarily at interior domains with folding propensity near to the fibril core (10), similar to the flickering β-sheet formation among NTD residues ∼30–60 and the NAC displayed by computation (24,27), while dynamical disorder in the flanking terminal regions is maintained. This is the compaction 1 process, represented by the rise in *W*_s_ (hydration region among compressing low-mobility protein regions) and corresponding decrease in *W*_f_ (dynamically disordered protein regions). At the end of the compaction 1 process, the distal subregions of the NTD (residues ∼30–60) fold onto the NAC region, forming stable β-sheet tertiary structure that extends radially from the cross-β interaction region in each protein layer, as observed in cryo-EM structures of α-synuclein fibrils (8,10,45). The hydration layer that forms around the surface of the new, stable tertiary structure extends along the fibril axis, following the fibril quaternary structure. The observed continuity at the kink in the log(*W*_s_/*W*_f_) dependence on *T* ^−1^ suggests that the water in the hydration region and the volume it occupied previously within the now-folded protein are comparable. The hydration water is stabilized against migration to the ice boundary by the relatively strong hydrogen bonding characteristic of water in hydration layers (46). The enveloping dynamically disordered CTD (and proximal NTD) attenuate ice boundary confinement of the hydration region, analogous to the effect of the aqueous-cryosolvent mesodomain around the hydration layer of globular proteins (20,41). In fact, the control (–DMSO) sample *E*_a,s_ (12 kcal/mol) and log(*τ*_c,s,0_) (−19) values are comparable with values for the hydration layer around folded, globular proteins under moderate confinement (11–13 kcal/mol; −20 to −19) (20). The consolidation of tertiary structure that caps the folding process, and the reorganization of protein-associated solvent to form an extended surface hydration domain that is relatively free of protein intrusion, is proposed as the origin of the kink that marks the transformation of the system to the compaction 2 linear relations in the *T* ^−1^ plots (Fig. 6). The compaction 2 process corresponds to collapse of the remaining disordered terminal domains. In the lowest-*T* states, a mélange of compressed CTD and proximal NTD domains, abutted by the ice boundary and intruding crystalline ice veins, envelops the hydration layer and folded internal protein structure (Fig. 7).

The model in Fig. 7 is consistent with the distribution of hydrogen bonds and the configurational entropy of the compacting regions of protein and coupled solvent as dominant determinants of the thermodynamics of the compaction processes. For both compaction 1 and 2, the net Δ*H*<0 and Δ*S*<0 (Table S5) are consistent with formation of strong interactions and loss of configurational freedom in the collapse process (47). Further, compaction 1 in the high-*T* regime is enthalpically less favorable (−6.6 versus −19 kcal/mol) and entropically more favorable (−28 versus −78 cal/mol/K) than compaction 2 in the low-*T* regime. The Δ*S* difference is consistent with the compaction of the less dynamic, internal portion of the protein structure at high *T*, relative to the loss of dynamic disorder in the multiconfiguration terminal domains at low *T*. Enthalpically, compaction and the relocation of water from protein-coupled regions to the ice boundary involves formation of strong water-ice hydrogen bonds (Δ*H* ≈ −5.5 kcal/mol) relative to loss of fluid water-water (Δ*H* ≈ −5.0 kcal/mol) (48) and water-protein hydrogen bonds (Δ*H* ≈ −1 to −2 kcal/mol) (49). Weaker water-protein hydrogen bonds in the dynamically disordered domain, relative to the quasi-stable protein regions proposed to dominate the compaction 1 process, would contribute to a more negative Δ*H* for compaction 2 in the low-*T* regime.

### Modulation of confinement compaction by dimethylsulfoxide

The model for α-synuclein response to confinement in Fig. 7 provides a basis for interpretation of the influence of DMSO on the Arrhenius and van’t Hoff relations (Fig. 6 *B* and *D*; parameter values; Table S5) for the compaction process. DMSO addition does not significantly alter the proportions of the spin probe-accessible hydration (slow component) and dynamically disordered (fast component) phases in the high-*T* regime (250–260 K), as shown by the common log(*W*_s_/*W*_f_) values (Fig. 6) or individual *W*_s_, *W*_f_ values (Fig. 3). This is consistent with infusion of both phases by DMSO, with water replacement, in the high-*T* range. The 1× and 3× DMSO conditions successively shift the pattern of the *T* ^−1^ dependence of log(*W*_s_/*W*_f_) to lower *T* values in approximately 5-K increments, signifying a perpetuation of the fast component to lower *T* values (Fig. 6 *D*). The shifts are caused by the fluidizing, cryosolvent effect of DMSO on low-*T* aqueous phases, which arises generally from the disruption of water structure (36,50). In greater detail, upon *T* decrease through compaction 1, the 1× and 3× DMSO conditions show successive decreases in the magnitudes of favorable Δ*H* and unfavorable Δ*S* (decreasing slope, increasing *y*-intercept, respectively, in Fig. 6 *D*), consistent with strong interactions of DMSO with water (51) and the exposed peptide regions (44), which together favor the disordered protein state. The patterns of *T* ^−1^ dependence for log*τ*_c,s_ and log*τ*_c,f_ with addition of 1× and 3× DMSO shift in concert with the log(*W*_s_/*W*_f_) pattern (same *T* value for the kink between compaction 1 and 2 regimes; Fig. 6 *B*). The values of *E*_a_ and log*τ*_c,0_ (includes activation entropy contribution) change little for the fast component with increasing DMSO, suggesting a strong role for protein actuation of dynamics in the disordered region. The formation of the stable hydration layer that precedes compaction 2 is predicted to preferentially exclude (43,44) DMSO from the hydration region (20), implying that DMSO resides principally in the dynamically disordered phase for compaction 2. This is consistent with the *T* dependence of *E*_a,s_ and log*τ*_c,0,s_ for the slow component in compaction 1 (presence of DMSO in weak hydration phase) and comparable values for *E*_a,s_ and log*τ*_c,0,s_ in compaction 2 (depletion of DMSO in strong hydration phase). Overall, the effects of DMSO on the phase equilibria (Δ*H*, Δ*S*) and spin probe mobility determinants (*E*_a_, log*τ*_c,0_) support the model and show how an excess of a small organic molecule can alter the protein-coupled solvent dynamics in α-synuclein fibrils.

### Thermal hysteresis arises from changes in dynamical cooperativity of terminal domains introduced by confinement

The *T* cycling experiment (Figs. 4 and 5) shows that hysteresis in fibrillar α-synuclein arises during the compaction 2 process, and that it accumulates incrementally. We propose that the hysteresis originates in the property of cooperativity in the dynamics of the interacting terminal domains in the fibril. In the fluid state at elevated *T* values, interactions among the terminal domains create an extended, dynamically disordered region along the length of the fibril that is maintained with the decreasing volume of the disordered region with decreasing *T* (panels 1–5, Fig. 7). However, beginning during compaction 2 and reaching maximum extent at the lowest *T* values (panel 6, Fig. 7), ice boundary confinement and ice intrusion between individual terminal domains lead to effective isolation of the terminal domains and, thus, quenching of the cooperative phase dynamics. Upon increase in *T* from the low-*T* limit, the isolation of terminal domains enforced by the boundary and intruding ice maintains the isolated state to higher temperatures. Additional thermal energy is required to fluidize and free the terminal domains, so that they can resume the interactions that characterize cooperative dynamics. Added DMSO relieves the frustration of dynamics resumption with increasing *T* by thwarting water crystallization, particularly in the ice intrusion zones and region surrounding the collapsed protein structure (19,20,41). The model is consistent with the absence of hysteresis under the same conditions in monomeric α-synuclein (11), which does not have the cooperative interprotein terminal domain interactions and longitudinally extended hydration structure. The model is also consistent with proposals that coupling of changes in solvation structure and material deformation underly thermal hysteresis in protein (52) and soft matter (53,54) systems.

A consequence of the hysteresis is bistability in the α-synuclein fibril protein and coupled solvent states, in the hysteresis *T* interval. Dependent upon thermal history, defined as the direction of approach to the interval by decreasing *T* (high *T* form) or increasing *T* (low *T* form), two forms of nominally comparable stability exist (model depiction of hysteresis and bistability, Fig. S5). The stabilities of the high-*T* and low-*T* forms are endowed by higher entropy and lower enthalpy, respectively. The phenomenon of bistability suggests that the fibril structure and dynamical state could be modulated or switched in vivo by high levels of confinement or crowding.

## Conclusions

The protein-coupled solvent dynamics and phase proportions reported by the EPR spin probe log*τ*_c_ and *W* parameters, respectively, provide a vivid portrayal of the physical properties and mechanical features of α-synuclein fibrils and their response to confinement. The ice boundary confinement induces structural and dynamical reorganization of the α-synuclein protein and coupled solvent, through the sequential processes, compaction 1 (proposed collapse of the distal NTD onto the NAC region), protein tertiary structure consolidation and stable hydration layer formation at the fibril core, and compaction 2 (collapse and restriction of the CTD and proximal NTD), with lowering *T*. Compaction 1 is likely to be accessible to in vivo volume exclusion by confinement and crowding (13). Indeed, the extrapolated Δ*G* for compaction at 37°C is relatively small, at 2 kcal/mol, suggesting that fibrils occupy a partially compacted state in vivo. Compaction 2 and the bistable states of fibril structure and dynamics revealed by the thermal hysteresis are predicted to be less readily achieved in vivo (Δ*G* ≈ 6 kcal/mol at 37°C). The results for DMSO show that small molecules can freely permeate the dynamically disordered domains and modify their physical properties. Quantification of fibril structural transitions and changes in associated protein-coupled solvent dynamics in terms of equilibrium (Δ*H*, Δ*S*) and kinetic (*E*_a_, log*τ*_c,0_) parameters leads to a detailed molecular mechanistic model. The approach of varied confinement offers a robust platform for quantitative assessment of drug-fibril interactions (for example, through differences introduced by the drug in Δ*H* and Δ*S* parameters) and drug design based on both classical tight-binding, complementary structure-based (55) and recently proposed distributed-binding, entropically based (34) and ensemble (35) paradigms. Future spin probe EPR experiments will address the mechanism of drug interactions with fibrillar, and monomeric and oligomeric (11,12), α-synuclein forms, as well as other pathologic IDPs.

## Data and code availability

Data supporting the findings of this study are tabulated in the supporting material. The custom code and methodology used to simulate EPR spectra are presented in Ref. (22) and are available upon request.

## Acknowledgments

Research reported in this publication was supported by the 10.13039/100000057National Institute of General Medical Sciences (R01 GM142113) of the 10.13039/100000002National Institutes of Health. The purchase of the Bruker E500 EPR spectrometer was funded by the 10.13039/100000097National Center for Research Resources of the 10.13039/100000002National Institutes of Health (RR 17767) and by 10.13039/100006939Emory University. The content is solely the responsibility of the authors and does not necessarily represent the official views of the National Institutes of Health.

## Author contributions

K.L.W. and K.W. conceived the article, analyzed EPR results, and performed writing, review, and editing. K.L.W. prepared EPR samples and performed EPR data acquisition, data reduction, and EPR simulations. K.W. acquired funding.

## Declaration of interests

The authors declare no competing interests.

## Supporting Material

Document S1. Figures S1–S5 and Tables S1–S5

Document S2. Article plus Supporting Material
