# Supplementary material for: Protein structure and coupled solvent dynamics in α-synuclein fibrils under controlled confinement
Source: Biophys J. 2026 Jan 29;125(5):1325–35. doi: 10.1016/j.bpj.2026.01.038 (PMC13290376; doi:10.1016/j.bpj.2026.01.038)
Supplement: Document S1. Figures S1–S5 and Tables S1–S5 [file mmc1.pdf]

Biophysical Journal, Volume *125*

Supplemental information

Protein structure and coupled solvent dynamics in α-synuclein fibrils under controlled confinement

Katie Lynn Whitcomb and Kurt Warncke

Protein Structure and Coupled Solvent Dynamics in α-Synuclein Fibrils under Controlled Confinement

Katie Lynn Whitcomb and Kurt Warncke

Department of Physics, Emory University, Atlanta, Georgia, 30322

Supporting Material

AUTHOR INFORMATION

Corresponding Author

Kurt Warncke - *Department of Physics, Emory University, Atlanta, Georgia 30322, United States*; orcid.org/0000-0002-3587-3720; Email: kwarncke@physics.emory.edu

Author

Katie Lynn Whitcomb - *Department of Physics, Emory University, Atlanta, Georgia 30322, United States*; orcid.org/0000-0003-4812-5438

Table of Contents

Supporting Figures

Page

Figure S1. Temperature dependence of the TEMPOL EPR spectrum in the presence of α-synuclein fibrils in the absence and presence of DMSO for collection of spectra in the direction of decreasing sequential temperature (*T*) change, and overlaid two-component EPR simulations. S-4

Figure S2. Circular dichroism (CD) spectroscopy of α-synuclein fibrils in the absence and presence of varying amounts of added DMSO. S-5

Figure S3. Depiction of the *T* cycling experiment. S-6

Figure S4. Temperature cycling dependence of the TEMPOL EPR spectra and overlaid simulations for fibrillar α-synuclein, for decreasing *T*, followed by increasing *T*. S-7

Figure S5. Depiction of the origin of thermal hysteresis and bistability in α-synuclein fibrils under *T*-controlled confinement. S-8

Supporting Tables

Table S1. Mean logτ_c and *W* values at different *T* values for α-synuclein fibrils in the absence of DMSO. S-9

Table S2. Mean logτ_c and *W* values at different *T* values for α-synuclein fibrils in the presence of 1× DMSO. S-10

Table S3. Mean logτ_c and W values at different T values for α-synuclein fibrils in the presence of 3× DMSO.
S-11

Table S4. Mean logτ_c and W values at different T values for α-synuclein fibrils for the recycling hysteresis experiment.
S-12

Table S5. Fitting parameters from Arrhenius and van't Hoff analysis of α-synuclein fibrils in the absence and presence of DMSO.
S-17

Supporting Figures

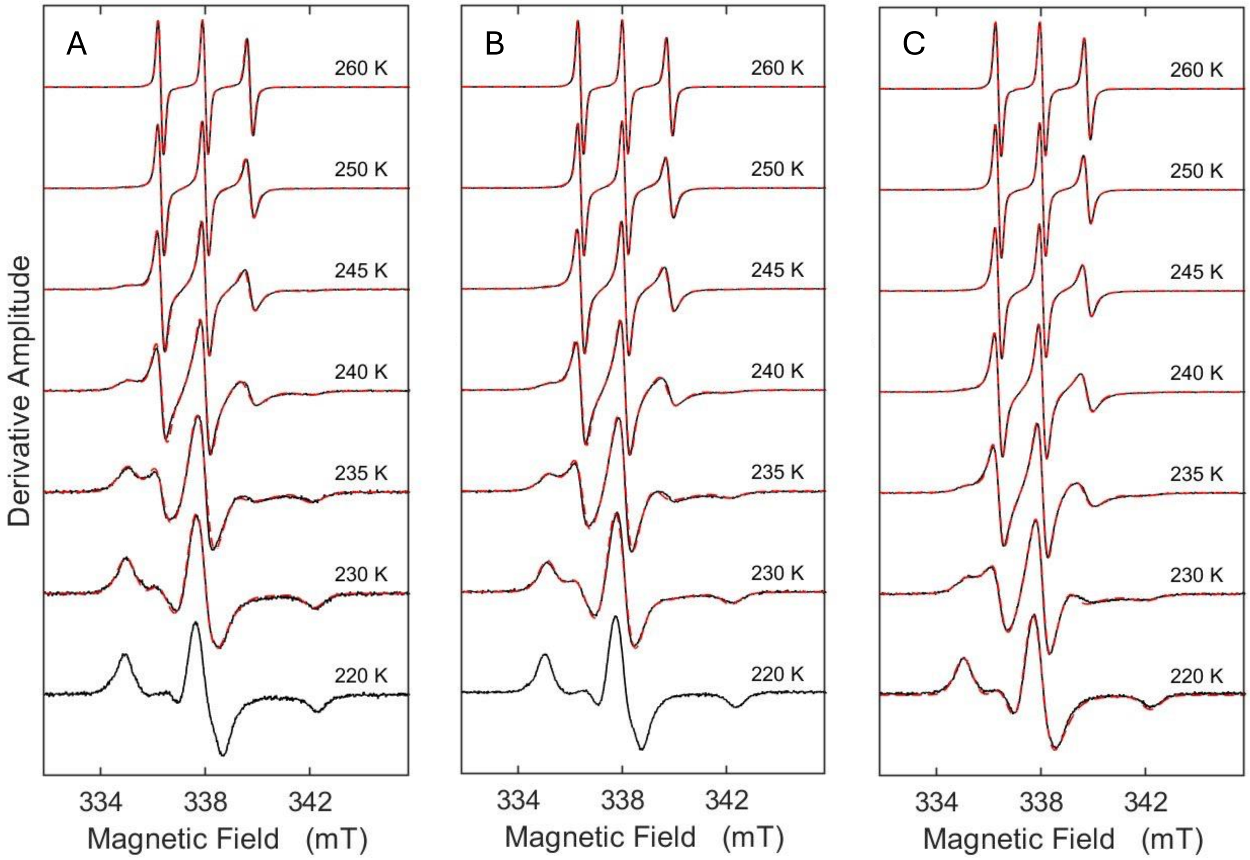

Figure S1. Temperature dependence of the TEMPOL EPR spectrum in the presence of α-synuclein fibrils for collection of spectra in the direction of decreasing sequential temperature change (black), overlaid with two-component EPR simulations (red). (A) In the abence of DMSO. (B) In the presence added DMSO, 1×. (C) In the presence of added DMSO, 3×.

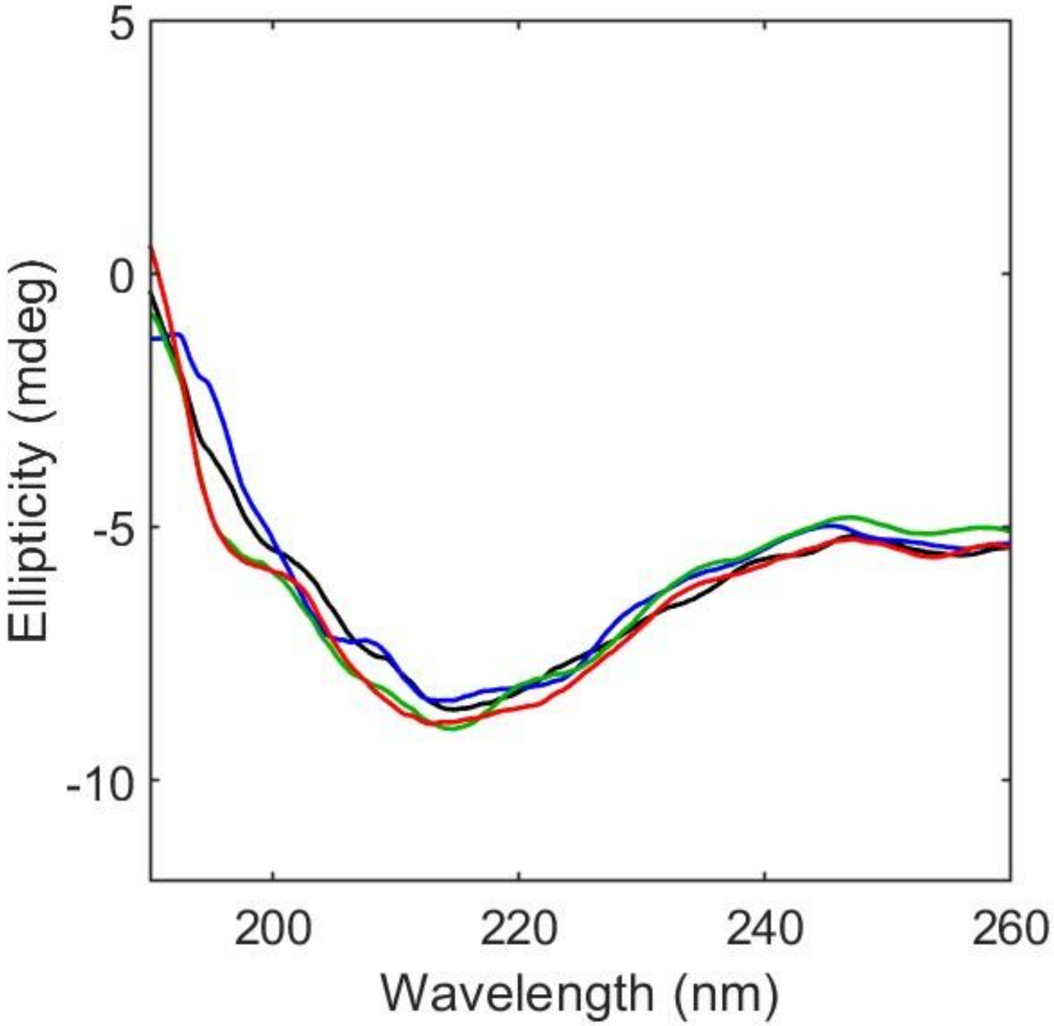

Figure S2. Circular dichroism (CD) spectroscopy of α-synuclein fibrils (no DMSO, control, black; 1× DMSO, red; 3× DMSO, green; 5× DMSO.

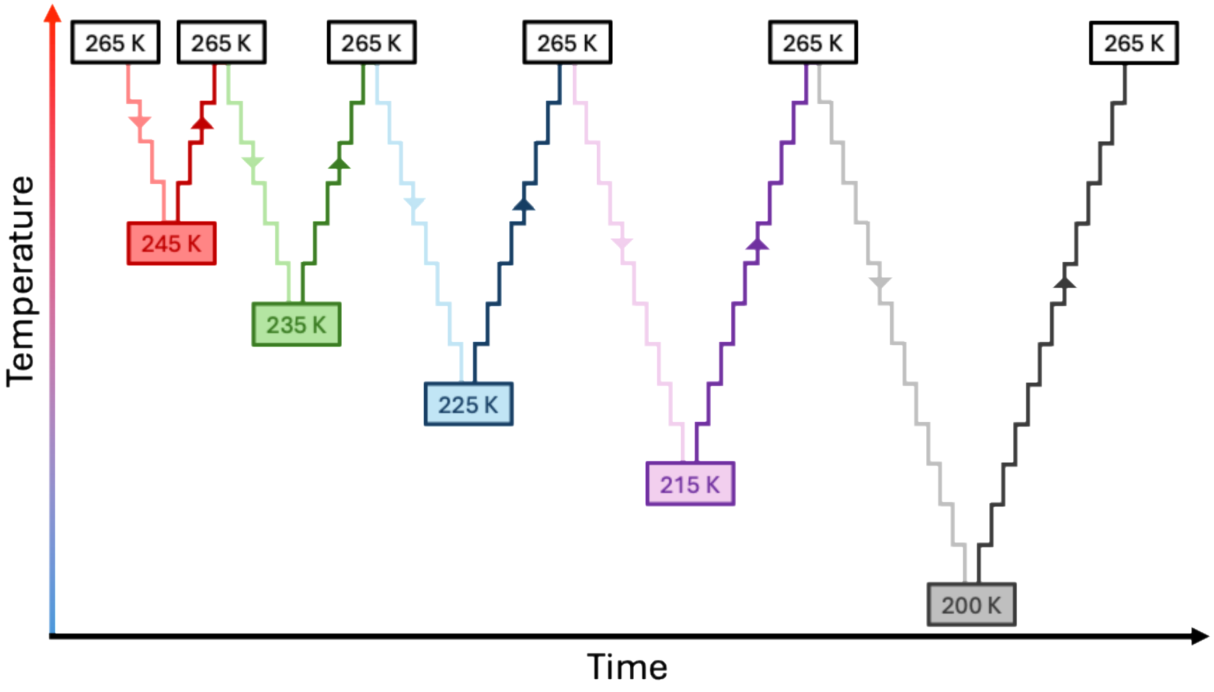

Figure S3. Depiction of the temperature (*T*) cycling experiment. The experiment is designed to determine the *T* range over which thermal hysteresis originates. The single EPR sample is initially held at 265 K (left, top), and then the *T* value is decreased in 5 K increments (pink path), with spectrum acquisition following a hold time of 300 s (5 min) at each temperature value. Upon reaching the terminal low *T* value, the *T* is increased in 5 K increments (red path), returning to 265 K. This pattern is repeated, for each descending terminal low *T* value, from left to right (increasing time). Each vertical line segment represents a *T* change of magnitude 5 K, and each horizontal line segment represents a time interval of 300 s plus the 80 s required for aquisition of four spectra, for averaging.

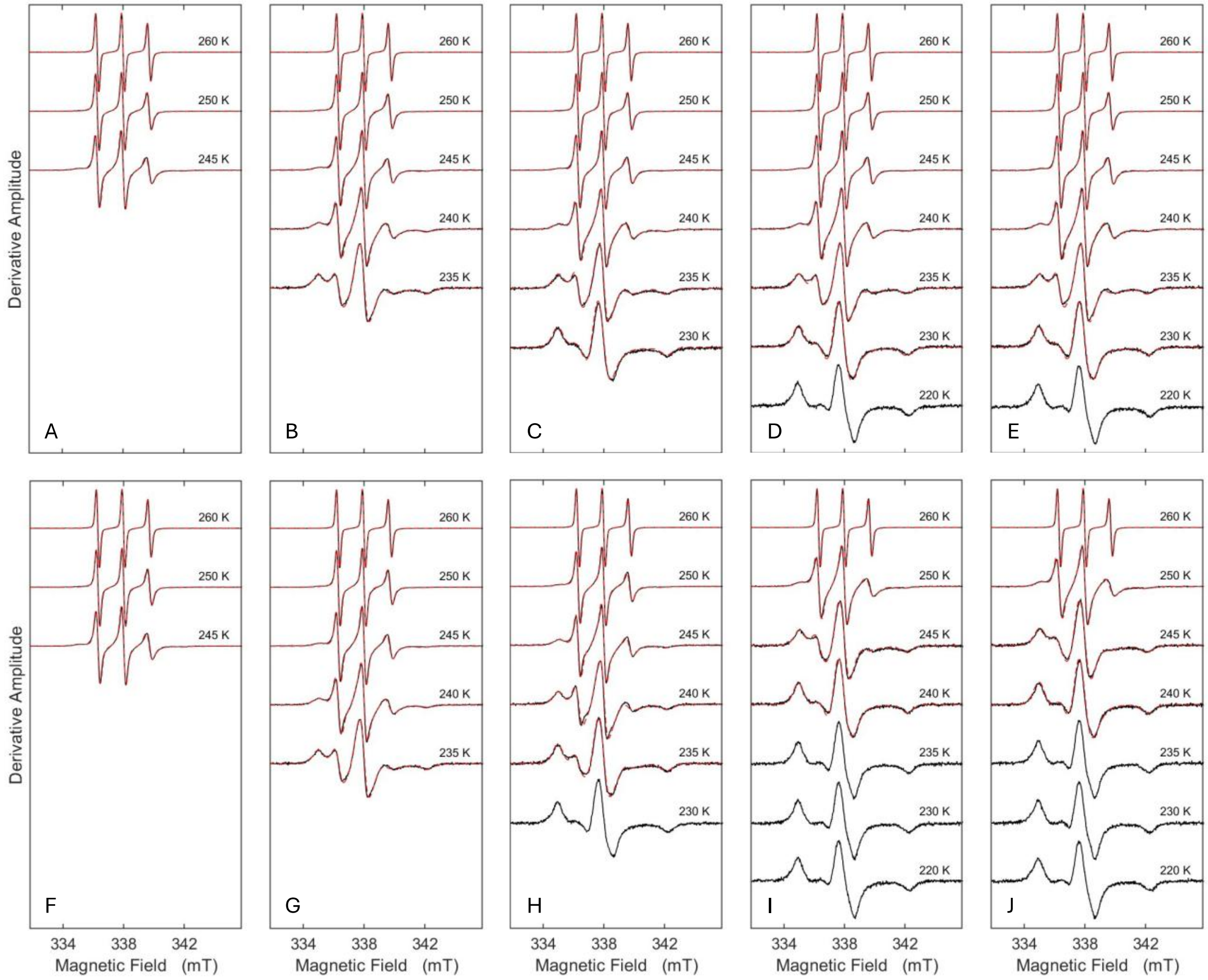

Figure S4. Temperature cycling dependence of the TEMPOL EPR spectra (black) and overlaid simulations (red) for fibrillar α-synuclein, for decreasing *T*, followed by increasing *T*. Spectrum cycles start from 265 K with spectrum collection in the direction of decreasing *T* (Panels A - E), and decrease to the base *T*, before return to 265 K with increasing *T* (Panels F - J). Spectra with no overlaid simulation correspond to rigid limit, $\log \tau_c < 7.0$ for both slow and fast components.

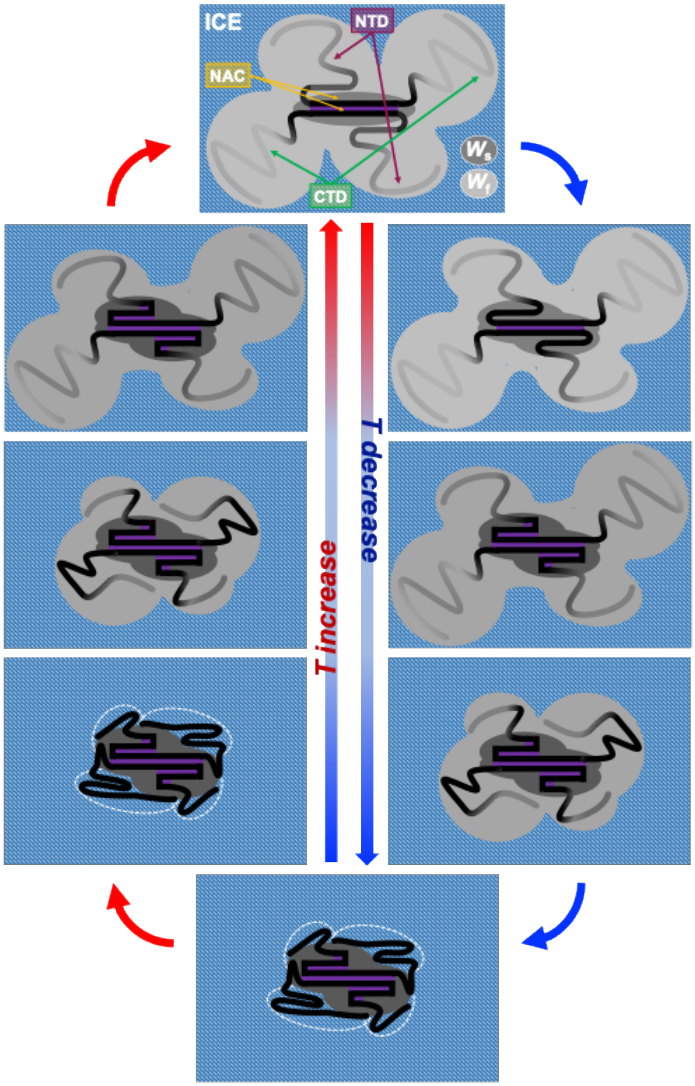

Figure S5. Depiction of the origin of thermal hysteresis and bistability in α-synuclein fibrils under *T*-controlled confinement. The depiction is based on the model representation, that is described in detail in text Figure 7. Hysteresis arises from compaction, ice intrusion, and eventual isolation of terminal domain segments in each layer of the fibril with decreasing *T* (right side) to form the non-interacting, statically disordered regions, as depicted within the white dashed curves in the bottom panel. The frustrated cooperative inter-layer dynamical interactions lead to promotion of the low *T* state to higher *T*, setting up bistability. Bistability is shown by the presence of the low *T*, low enthalpy (left) and high *T*, high entropy (right) states at the same *T* value (same vertical position). At elevated *T* values, cooperative dynamics recommence, leading to population of the common high *T* state (top panel).

Supporting Tables

Table S1. Mean logτ_c and W values at different T values for α-synuclein fibrils in the absence of DMSO (control), for data collection in the direction of increasing and decreasing T. Values of $\tau_{c,s}$ and $\tau_{c,f}$ are referenced to the τ_c value of 1 s.

Increasing T

T **(K)**	**log $\tau_{c,s}$ (s)**	W_s	**log $\tau_{c,f}$ (s)**	W_f
240	-6.86 ± 0.20	0.78 ± 0.04	-8.06 ± 0.06	0.22 ± 0.04
245	-7.33 ± 0.06	0.75 ± 0.01	-8.47 ± 0.00	0.25 ± 0.01
250	-7.79 ± 0.04	0.47 ± 0.01	-8.95 ± 0.02	0.53 ± 0.01
255	-8.84 ± 0.05	0.37 ± 0.00	-9.46 ± 0.01	0.63 ± 0.00
260	-9.48 ± 0.04	0.33 ± 0.02	-9.74 ± 0.01	0.67 ± 0.02
265	-9.78 ± 0.00	0.32 ± 0.01	-9.98 ± 0.00	0.68 ± 0.01

Decreasing T

T **(K)**	**log $\tau_{c,s}$ (s)**	W_s	**log $\tau_{c,f}$ (s)**	W_f
230	-7.23 ± 0.01	0.90 ± 0.02	-8.49 ± 0.11	0.10 ± 0.02
235	-7.51 ± 0.00	0.74 ± 0.01	-8.61 ± 0.01	0.26 ± 0.01
240	-7.70 ± 0.04	0.55 ± 0.02	-8.78 ± 0.02	0.45 ± 0.02
245	-7.88 ± 0.01	0.43 ± 0.00	-9.03 ± 0.02	0.57 ± 0.00
250	-8.49 ± 0.06	0.36 ± 0.01	-9.29 ± 0.02	0.64 ± 0.01
255	-9.12 ± 0.10	0.29 ± 0.01	-9.53 ± 0.01	0.71 ± 0.01
260	-9.44 ± 0.02	0.26 ± 0.01	-9.77 ± 0.01	0.74 ± 0.01
265	-9.81 ± 0.03	0.30 ± 0.00	-9.96 ± 0.00	0.70 ± 0.00

Table S2. Mean logτ_c and W values at different T values for α-synuclein fibrils in the presence of 1× DMSO, for data collection in the direction of increasing T, and for data collection in the direction of decreasing T. Values of $\tau_{c,s}$ and $\tau_{c,f}$ are referenced to the τ_c value of 1 s.

Increasing T:

T (K)	log $\tau_{c,s}$ (s)	W_s	log $\tau_{c,f}$ (s)	W_f
230	-6.88 ± 0.09	0.87 ± 0.02	-8.03 ± 0.22	0.13 ± 0.02
235	-7.24 ± 0.03	0.76 ± 0.02	-8.40 ± 0.01	0.24 ± 0.02
240	-7.54 ± 0.03	0.65 ± 0.06	-8.51 ± 0.02	0.35 ± 0.06
245	-7.95 ± 0.04	0.47 ± 0.03	-8.82 ± 0.02	0.53 ± 0.03
250	-8.72 ± 0.08	0.41 ± 0.04	-9.20 ± 0.01	0.59 ± 0.04
255	-9.26 ± 0.02	0.31 ± 0.01	-9.52 ± 0.01	0.69 ± 0.01
260	-9.55 ± 0.02	0.30 ± 0.01	-9.77 ± 0.00	0.70 ± 0.01

Decreasing T:

T (K)	log $\tau_{c,s}$ (s)	W_s	log $\tau_{c,f}$ (s)	W_f
225	-7.09 ± 0.06	0.89 ± 0.03	-8.35 ± 0.15	0.11 ± 0.03
230	-7.40 ± 0.02	0.76 ± 0.02	-8.46 ± 0.01	0.24 ± 0.02
235	-7.54 ± 0.08	0.58 ± 0.03	-8.53 ± 0.01	0.42 ± 0.03
240	-7.93 ± 0.05	0.46 ± 0.02	-8.79 ± 0.02	0.54 ± 0.02
245	-8.32 ± 0.04	0.39 ± 0.01	-9.06 ± 0.01	0.61 ± 0.01
250	-8.93 ± 0.00	0.34 ± 0.01	-9.30 ± 0.00	0.66 ± 0.01
255	-9.24 ± 0.02	0.27 ± 0.01	-9.55 ± 0.01	0.73 ± 0.01
260	-9.54 ± 0.03	0.28 ± 0.01	-9.78 ± 0.00	0.72 ± 0.01

Table S3. Mean logτ_c and W values at different T values for α-synuclein fibrils in the presence of 3× DMSO, for data collection in the direction of increasing T, and for data collection in the direction of decreasing T. Values of $\tau_{c,s}$ and $\tau_{c,f}$ are referenced to the τ_c value of 1 s.

Increasing T:

T **(K)**	**log $\tau_{c,s}$ (s)**	W_s	**log $\tau_{c,f}$ (s)**	W_f
220	-7.33 ± 0.09	0.67 ± 0.07	-8.18 ± 0.07	0.33 ± 0.07
225	-7.32 ± 0.04	0.50 ± 0.01	-8.31 ± 0.03	0.50 ± 0.01
230	-7.83 ± 0.11	0.47 ± 0.01	-8.54 ± 0.04	0.53 ± 0.01
235	-7.94 ± 0.12	0.42 ± 0.01	-8.75 ± 0.03	0.58 ± 0.01
240	-8.34 ± 0.06	0.47 ± 0.01	-8.95 ± 0.02	0.53 ± 0.01
245	-8.59 ± 0.01	0.43 ± 0.01	-9.10 ± 0.00	0.57 ± 0.01
250	-9.10 ± 0.01	0.34 ± 0.01	-9.35 ± 0.00	0.66 ± 0.01
255	-9.44 ± 0.05	0.34 ± 0.02	-9.60 ± 0.00	0.66 ± 0.02
260	-9.64 ± 0.01	0.35 ± 0.00	-9.83 ± 0.01	0.65 ± 0.00

Decreasing T:

T **(K)**	**log $\tau_{c,s}$ (s)**	W_s	**log $\tau_{c,f}$ (s)**	W_f
220	-7.13 ± 0.04	0.79 ± 0.07	-8.11 ± 0.06	0.21 ± 0.07
225	-7.35 ± 0.04	0.64 ± 0.01	-8.34 ± 0.00	0.36 ± 0.01
230	-7.72 ± 0.03	0.53 ± 0.01	-8.46 ± 0.02	0.47 ± 0.01
235	-7.96 ± 0.02	0.40 ± 0.01	-8.72 ± 0.03	0.60 ± 0.01
240	-8.43 ± 0.00	0.40 ± 0.01	-8.97 ± 0.01	0.60 ± 0.01
245	-8.76 ± 0.00	0.34 ± 0.01	-9.18 ± 0.00	0.66 ± 0.01
250	-9.15 ± 0.02	0.28 ± 0.01	-9.40 ± 0.01	0.72 ± 0.01
255	-9.39 ± 0.01	0.28 ± 0.02	-9.62 ± 0.00	0.72 ± 0.02
260	-9.66 ± 0.03	0.33 ± 0.00	-9.82 ± 0.00	0.67 ± 0.00

Table S4. Mean logτ_c and W values at different T values for α-synuclein fibrils for the recycling hysteresis protocol. Data collection proceeded in the direction of decreasing T to different base T values, followed by data collection in the direction of increasing T. The same sample was used for the entire series, starting with the 245 K cycle, and ending with the 200 K cycle. The base T values correspond to: 245, 235, 225, 215, and 200 K. Values of $\tau_{c,s}$ and $\tau_{c,f}$ are referenced to the τ_c value of 1 s.

245 K base *T*

Decreasing T:

T **(K)**	**log $\tau_{c,s}$ (s)**	W_s	**log $\tau_{c,f}$ (s)**	W_f
245	-8.00 ± 0.04	0.37 ± 0.04	-9.09 ± 0.04	0.63 ± 0.04
250	-8.67 ± 0.02	0.32 ± 0.02	-9.36 ± 0.02	0.68 ± 0.02
255	-9.06 ± 0.02	0.27 ± 0.02	-9.59 ± 0.02	0.73 ± 0.02
260	-9.32 ± 0.01	0.23 ± 0.01	-9.82 ± 0.02	0.77 ± 0.01
265	-9.69 ± 0.01	0.23 ± 0.01	-10.08 ± 0.09	0.77 ± 0.01

Increasing T:

T **(K)**	**log $\tau_{c,s}$ (s)**	W_s	**log $\tau_{c,f}$ (s)**	W_f
245	-7.97 ± 0.07	0.38 ± 0.02	-9.10 ± 0.05	0.62 ± 0.02
250	-8.66 ± 0.26	0.34 ± 0.02	-9.34 ± 0.02	0.66 ± 0.02
255	-9.04 ± 0.13	0.26 ± 0.02	-9.57 ± 0.02	0.74 ± 0.02
260	-9.31 ± 0.10	0.23 ± 0.01	-9.82 ± 0.02	0.77 ± 0.01
265	-9.57 ± 0.06	0.22 ± 0.03	-10.03 ± 0.03	0.78 ± 0.03

235 K base *T*

Decreasing T:

T (K)	log $\tau_{c,s}$ (s)	W_s	log $\tau_{c,f}$ (s)	W_f
235	-7.35 ± 0.13	0.68 ± 0.07	-8.59 ± 0.06	0.32 ± 0.07
240	-7.65 ± 0.12	0.50 ± 0.05	-8.84 ± 0.02	0.50 ± 0.05
245	-7.88 ± 0.08	0.37 ± 0.06	-9.11 ± 0.03	0.63 ± 0.06
250	-8.59 ± 0.16	0.33 ± 0.02	-9.35 ± 0.02	0.67 ± 0.02
255	-9.03 ± 0.04	0.26 ± 0.02	-9.60 ± 0.02	0.74 ± 0.02
260	-9.25 ± 0.05	0.23 ± 0.02	-9.83 ± 0.02	0.77 ± 0.02
265	-9.60 ± 0.07	0.22 ± 0.02	-10.08 ± 0.06	0.78 ± 0.02

Increasing T:

T (K)	log $\tau_{c,s}$ (s)	W_s	log $\tau_{c,f}$ (s)	W_f
235	-7.38 ± 0.05	0.69 ± 0.04	-8.59 ± 0.04	0.31 ± 0.04
240	-7.54 ± 0.13	0.57 ± 0.05	-8.81 ± 0.02	0.43 ± 0.05
245	-7.85 ± 0.02	0.46 ± 0.05	-9.04 ± 0.00	0.54 ± 0.05
250	-8.46 ± 0.19	0.39 ± 0.02	-9.29 ± 0.01	0.61 ± 0.02
255	-8.99 ± 0.14	0.28 ± 0.02	-9.52 ± 0.02	0.72 ± 0.02
260	-9.23 ± 0.06	0.23 ± 0.01	-9.83 ± 0.02	0.77 ± 0.01
265	-9.58 ± 0.11	0.21 ± 0.01	-10.06 ± 0.06	0.79 ± 0.01

<u>225 K base *T*</u>

Decreasing T:

T (K)	log $\tau_{c,s}$ (s)	W_s	log $\tau_{c,f}$ (s)	W_f
230	-7.16 ± 0.04	0.85 ± 0.06	-8.43 ± 0.10	0.15 ± 0.06
235	-7.35 ± 0.13	0.69 ± 0.08	-8.59 ± 0.06	0.31 ± 0.08
240	-7.65 ± 0.12	0.50 ± 0.06	-8.84 ± 0.02	0.50 ± 0.06
245	-7.88 ± 0.08	0.38 ± 0.03	-9.11 ± 0.03	0.62 ± 0.03
250	-8.59 ± 0.16	0.33 ± 0.02	-9.35 ± 0.02	0.67 ± 0.02
255	-9.03 ± 0.04	0.27 ± 0.02	-9.60 ± 0.02	0.73 ± 0.02
260	-9.25 ± 0.05	0.22 ± 0.01	-9.83 ± 0.02	0.78 ± 0.01
265	-9.60 ± 0.07	0.22 ± 0.01	-10.08 ± 0.06	0.78 ± 0.01

Increasing T:

T (K)	log $\tau_{c,s}$ (s)	W_s	log $\tau_{c,f}$ (s)	W_f
235	-7.20 ± 0.10	0.82 ± 0.07	-8.64 ± 0.13	0.18 ± 0.07
240	-7.35 ± 0.09	0.68 ± 0.08	-8.66 ± 0.07	0.32 ± 0.08
245	-7.76 ± 0.05	0.59 ± 0.08	-8.93 ± 0.03	0.41 ± 0.08
250	-8.17 ± 0.27	0.45 ± 0.06	-9.17 ± 0.02	0.55 ± 0.06
255	-8.87 ± 0.13	0.31 ± 0.03	-9.48 ± 0.04	0.69 ± 0.03
260	-9.25 ± 0.03	0.23 ± 0.01	-9.83 ± 0.02	0.77 ± 0.01
265	-9.56 ± 0.00	0.22 ± 0.02	-10.07 ± 0.04	0.78 ± 0.02

<u>215 K base *T*</u>

Decreasing T:

T (K)	log $\tau_{c,s}$ (s)	W_s	log $\tau_{c,f}$ (s)	W_f
230	-7.21 ± 0.03	0.85 ± 0.06	-8.46 ± 0.11	0.15 ± 0.06
235	-7.34 ± 0.06	0.70 ± 0.07	-8.56 ± 0.01	0.30 ± 0.07
240	-7.59 ± 0.13	0.52 ± 0.04	-8.83 ± 0.03	0.48 ± 0.04
245	-7.90 ± 0.09	0.39 ± 0.04	-9.11 ± 0.04	0.61 ± 0.04
250	-8.85 ± 0.16	0.33 ± 0.02	-9.36 ± 0.02	0.67 ± 0.02
255	-9.01 ± 0.04	0.27 ± 0.02	-9.60 ± 0.02	0.73 ± 0.02
260	-9.19 ± 0.04	0.23 ± 0.02	-9.83 ± 0.01	0.77 ± 0.02
265	-9.54 ± 0.03	0.22 ± 0.02	-10.07 ± 0.05	0.78 ± 0.02

Increasing T:

T (K)	log $\tau_{c,s}$ (s)	W_s	log $\tau_{c,f}$ (s)	W_f
240	-7.12 ± 0.13	0.78 ± 0.05	-8.36 ± 0.12	0.22 ± 0.05
245	-7.56 ± 0.04	0.71 ± 0.12	-8.60 ± 0.04	0.29 ± 0.12
250	-7.89 ± 0.04	0.47 ± 0.05	-8.95 ± 0.09	0.53 ± 0.05
255	-8.84 ± 0.19	0.31 ± 0.03	-9.45 ± 0.05	0.69 ± 0.03
260	-9.22 ± 0.02	0.23 ± 0.02	-9.83 ± 0.01	0.77 ± 0.02
265	-9.48 ± 0.10	0.21 ± 0.02	-10.06 ± 0.04	0.79 ± 0.02

<u>200 K base *T*</u>

Decreasing T:

T (K)	log $\tau_{c,s}$ (s)	W_s	log $\tau_{c,f}$ (s)	W_f
230	-7.21 ± 0.02	0.87 ± 0.04	-8.47 ± 0.05	0.13 ± 0.04
235	-7.44 ± 0.06	0.70 ± 0.07	-8.58 ± 0.05	0.30 ± 0.07
240	-7.62 ± 0.05	0.52 ± 0.05	-8.79 ± 0.03	0.48 ± 0.05
245	-7.88 ± 0.01	0.40 ± 0.04	-9.07 ± 0.05	0.60 ± 0.04
250	-8.63 ± 0.26	0.33 ± 0.02	-9.32 ± 0.04	0.67 ± 0.02
255	-9.06 ± 0.08	0.27 ± 0.02	-9.56 ± 0.04	0.73 ± 0.02
260	-9.36 ± 0.19	0.24 ± 0.01	-9.79 ± 0.03	0.76 ± 0.01
265	-9.72 ± 0.18	0.27 ± 0.06	-10.00 ± 0.04	0.73 ± 0.06

Increasing T:

T (K)	log $\tau_{c,s}$ (s)	W_s	log $\tau_{c,f}$ (s)	W_f
240	-7.11 ± 0.27	0.79 ± 0.07	-8.14 ± 0.24	0.21 ± 0.07
245	-7.40 ± 0.08	0.68 ± 0.14	-8.52 ± 0.08	0.32 ± 0.14
250	-7.93 ± 0.16	0.46 ± 0.05	-8.92 ± 0.10	0.54 ± 0.05
255	-8.88 ± 0.11	0.30 ± 0.04	-9.46 ± 0.04	0.70 ± 0.04
260	-9.33 ± 0.14	0.23 ± 0.02	-9.79 ± 0.03	0.77 ± 0.02
265	-9.68 ± 0.16	0.23 ± 0.02	-10.05 ± 0.06	0.77 ± 0.02

Table S5. Fitting parameters from Arrhenius and van't Hoff analysis for α-synuclein fibrils in the absence and presence of DMSO. Data is from the direction of decreasing T. The T range high corresponds to compaction 1, T range low to compaction 2. Mean values and standard deviations correspond to three independent measurements. Value of the Arrhenius plot y-intercept, $\log \tau_{c,0}$, is referenced to the τ_c value of 1 s. Categories are colored (shaded) by % DMSO to guide the eye.

Arrhenius Analysis						
**Relative % v/v DMSO**	**Component**	***T* range**	**E_a (kcal/mol)**	**Std. dev.**	**$\log \tau_{c,0}$**	**Std. dev.**
0	Slow	Low	11.9	0.7	-18.5	0.7
		High	31.0	0.8	-35.6	0.8
	Fast	Low	7.3	2.2	-10.0	9.7
		High	14.4	0.1	-21.9	0.1
1	Slow	Low	10.7	0.3	-17.5	0.4
		High	25.7	0.8	-31.3	0.6
	Fast	Low	6.7	1.5	-14.8	1.4
		High	14.2	0.4	-21.7	0.3
3	Slow	Low	13.6	0.3	-20.6	0.3
		High	20.9	0.0	-27.5	0.0
	Fast	Low	8.2	0.8	-16.3	0.8
		High	12.3	0.3	-20.1	0.3

van't Hoff Analysis					
**Relative % v/v DMSO**	***T* range**	**ΔH (kcal/mol)**	**Std. dev.**	**ΔS (cal/mol/K)**	**Std. dev.**
0	Low	-21.4	-3.1	-88.9	13.2
	High	-6.8	-0.4	-28.2	1.6
1	Low	-17.0	-2.5	-71.8	11.2
	High	-6.5	-0.1	-27.3	0.3
3	Low	-12.6	-4.7	-54.6	20.5
	High	-4.3	-0.7	-19.0	3.0
